# Experiences of Inspiratory Muscle Training and Physical Exercise in Adults with Post-COVID-19 Condition and Inspiratory Muscle Weakness: A Qualitative Study

**DOI:** 10.3390/jcm15197414

**Published:** 2026-09-24

**Authors:** Anna Törnberg, Anna Svensson-Raskh, Annie Svensson, Ulrika Holdar, Judith Bruchfeld, Elisabeth Rydwik, Malin Nygren-Bonnier

**Affiliations:** 1 Division of Physiotherapy, Department of Neurobiology, Care Sciences and Society, Karolinska Institutet, SE-141 83 Huddinge, Sweden; anna.svensson-raskh@ki.se (A.S.-R.); annie.svensson@ki.se (A.S.); elisabeth.rydwik@ki.se (E.R.); malin.nygren-bonnier@ki.se (M.N.-B.); 2 Medical Unit Allied Health Professionals, Women’s Health and Allied Health Professionals Theme, Karolinska University Hospital, SE-171 76 Stockholm, Sweden; ulrika.holdar@ki.se; 3 Division of Infectious Diseases, Department of Medicine Solna, Karolinska Institutet, SE-171 76 Stockholm, Sweden; judith.bruchfeld@ki.se; 4 Department of Infectious Diseases, Theme Emergency and Reparative Medicine, Karolinska University Hospital, SE-171 76 Stockholm, Sweden

**Keywords:** exercise, inspiratory muscle training, muscle weakness, post-acute COVID-19 syndrome, qualitative research, rehabilitation, respiratory muscles

## Abstract

**Background/Objectives:** Inspiratory muscle training and physical exercise may benefit individuals with post-COVID-19 condition (PCC), but little is known about how such interventions are experienced. This study aimed to describe experiences of an individualized intervention combining inspiratory muscle training and physical exercise among adults with PCC and inspiratory muscle weakness. **Methods:** A qualitative descriptive study using inductive content analysis was conducted at a tertiary-level PCC service in Sweden. Semi-structured individual interviews were conducted with 12 adults (11 women; median age 54 years) with PCC and inspiratory muscle weakness who had participated in a previous randomized controlled trial. **Results:** Participants entered the intervention with varying expectations and perceived it as a meaningful opportunity for self-directed action. They described adapting the intervention through learning, gradual progression, and individual adjustments in response to challenging symptoms and everyday demands as the intervention involved physical and emotional strain, symptom-related uncertainty, and practical challenges. Professional support and regular, flexible follow-up were perceived to support a sense of safety and continued engagement. Perceived outcomes ranged from partial improvements and renewed trust in their bodies to uncertainty, no perceived change, or setbacks. **Conclusions:** Adults with PCC and inspiratory muscle weakness who were able to participate described both benefits and challenges and often perceived the tailored intervention as a meaningful way to take an active role in rehabilitation while navigating persistent symptoms. Individualized delivery and professional support were perceived to support engagement while accommodating symptom fluctuations, and perceived effects ranged widely. These findings highlight the importance of tailored rehabilitation approaches that address the complex and fluctuating nature of PCC while providing flexible support.

## 1. Introduction

Post-COVID-19 condition (PCC) is characterized by a wide range of prolonged and often fluctuating symptoms and impairments that limit daily functioning in individuals following COVID-19 and pose substantial rehabilitation challenges [1,2,3]. Common features include fatigue, respiratory manifestations, post-exertional symptom exacerbation (PESE), reduced physical capacity, and cognitive impairments [1,4,5]. Among respiratory manifestations, dyspnea, altered breathing pattern, and inspiratory muscle weakness are common [1,4,6,7]. PCC manifestations can impact physical activity, work ability, mental health, and activities of daily life [2,3,5,8]. As no curative treatment currently exists, effective and safe rehabilitation is essential [1,2,9].

Rehabilitative interventions in PCC show promise, but individuals’ perspectives remain underexplored [1,2,8,10,11]. Although inspiratory muscle training (IMT) and physical exercise show beneficial effects for individuals with PCC and respiratory manifestations, including improvements in inspiratory muscle strength, physical capacity, and symptoms [2,8], outcome measures provide only a partial understanding of the rehabilitation process [10,11]. To gain a more comprehensive picture, it is essential to include the experiences of affected individuals [10,11]. Previous qualitative studies in PCC highlight that IMT is often perceived as helpful yet demanding, requiring discipline and individualized approaches with regular follow-up [12,13,14]. Similarly, physical activity and exercise are generally perceived as beneficial but require acceptance of physical limitations, consideration of factors such as PESE, and flexible, tailored rehabilitation approaches [15,16,17]. To date, no study has described experiences of an individualized intervention combining IMT and physical exercise in individuals with PCC. Addressing this knowledge gap is essential for advancing person-centered rehabilitation strategies and optimizing the design and delivery of exercise-based interventions for PCC.

Therefore, this study aimed to describe how adults with PCC and inspiratory muscle weakness experienced an eight-week individualized rehabilitative intervention consisting of IMT and physical exercise.

## 2. Materials and Methods

### 2.1. Study Design

A qualitative descriptive methodology was employed, underpinned by a constructivist perspective, to provide a broad, nuanced, and contextually grounded description of participants’ experiences [18,19]. The study adhered to the COREQ (Consolidated Criteria for Reporting Qualitative Research) guidelines to ensure methodological transparency and rigor (see Supplementary Materials) [20].

The study was approved by the Swedish Ethical Review Authority (Approval number: 2020-02149), and informed written consent was obtained from all participants. The study follows the Helsinki Declaration and was performed in accordance with Swedish law and regulations.

### 2.2. Setting and Participants

This study forms part of the ReCOV research project (Recovery and rehabilitation during and after COVID-19), which is described in detail elsewhere [7,21]. Participants in ReCOV were recruited from the specialized post-COVID outpatient clinic at Karolinska University Hospital in Stockholm, Sweden. The outpatient clinic is a tertiary-level, interprofessional service to which both adults previously hospitalised for COVID-19, and non-hospitalised individuals with suspected PCC and ≥50% ongoing sick leave or equivalent impaired functioning, are referred for clinical assessment.

Study participants were recruited from the intervention group in a preceding randomized controlled trial (RCT) [22]. The RCT evaluated an individualized intervention combining twice-daily inspiratory muscle training (IMT) with twice-weekly physical exercise compared with exercise alone, in adults with PCC and inspiratory muscle weakness. The RCT included adults with PCC according to the WHO definition and inspiratory muscle weakness, defined as maximal inspiratory pressure ≤ 80% of the lower limit of the 95% confidence interval of the predicted value [23,24]. Participants performed IMT using a handheld device (Threshold IMT, Philips Respironics, Murrysville, PA, USA or POWERbreathe K3, POWERbreathe International Ltd., Southam, UK), with training load individually prescribed according to inspiratory muscle strength and adjusted based on perceived exertion and the occurrence of PESE. Physical exercise included individually tailored aerobic, resistance, and mobility exercises, with intensity, volume, and progression adapted based on perceived exertion and the occurrence of PESE. Participants attended weekly physiotherapist follow-ups during the eight-week intervention and completed baseline and post-intervention assessments. The intervention was delivered by specialized physiotherapists in university-hospital or primary-care outpatient settings. IMT was home-based, whereas exercise sessions and follow-ups were conducted on-site or remotely (video or phone) according to preference. Further details of the intervention have been reported elsewhere [22].

Purposive sampling was applied, whereby all participants allocated to the intervention group who completed the RCT (*n* = 16) were invited to participate in individual interviews. This approach enabled the inclusion of participants with a range of experiences relevant to the study aim. Potential participants were contacted by phone by one of two authors (AS, UH), who also conducted the interviews. Information about participants included demographic data, medical history, and baseline clinical characteristics from the preceding RCT, all retrieved from medical records.

### 2.3. Data Collection

All participants were individually interviewed on one occasion in person, by telephone, or via video depending on their preference. The interviews took place between March 2022 and March 2024, following completion of the RCT. The interviews were conducted by physiotherapists (AS, *n* = 9; UH, *n* = 3) with clinical experience in rehabilitation and PCC; one interviewer had prior qualitative interviewing experience (UH). Neither interviewer had a pre-existing relationship with the participants or any involvement in the preceding RCT, and had no knowledge regarding participants’ adherence, clinical outcomes, or other study results at the time of the interviews.

A semi-structured interview guide (see Supplementary Materials) with open-ended questions was developed by two authors (MNB, AT), addressing participants’ experiences of intervention content, delivery, expectations, challenges, and impact. After the first two interviews, the guide was reviewed for relevance by three authors (MNB, AT, UH). No revisions were deemed necessary and both interviews were included in the final analysis. The interviews were audio-recorded and transcribed verbatim.

### 2.4. Data Analysis

The interview transcripts were analyzed using inductive content analysis, enabling a data-driven understanding of participants’ perspectives [25]. The analytic process followed the approach outlined by Vears and Gillam [25], with further guidance from Lindgren, Graneheim and Lundman on ensuring coherent abstraction levels, where categories express manifest content at a higher level than sub-categories [26]. The qualitative interview data were analyzed independently of adherence data and clinical outcomes collected in the preceding RCT.

To gain a comprehensive understanding of the data, all transcripts and corresponding audio recordings were read and listened to several times by one author (AT). In the first coding round, text segments relevant to the study aim (i.e., meaning units) were identified and organized into preliminary, broad content categories, consistent with the “big-picture coding” approach described by Vears and Gillam [25]. In the second coding round, all coded text within each preliminary content category was examined line by line, resulting in the development of codes, subcategories, and refinement of the content categories. Subcategories were continuously compared within and across categories and refined iteratively throughout the analysis process to ensure that they accurately reflected the data, resulting in a refined final coding scheme. While some codes captured experiences specifically related to IMT or physical exercise, subcategories and categories were developed to reflect participants’ experiences of the combined intervention, in line with the study aim. Examples illustrating the progression from meaning unit to code, subcategory, and content category are presented in Table 1. Content categories and subcategories were then synthesized and interpreted, focusing on the manifest content, to address the research aim [25]. Dedoose software (version 9.0.107) was used to support data management and organization of codes throughout the analysis process [27].

The first author (AT) conducted the initial coding of all interviews. To strengthen consistency and reflexivity, the last author (MNB) independently coded two of the interviews that had already been coded by AT. The two interviews were purposively selected to capture variation in participant experiences and perceptions of the intervention, including differences in perceived benefits, challenges, and overall experiences. Coding decisions and interpretations were then compared and discussed to support consistency and reflexivity in the analytical process. The independent coding showed a high degree of agreement regarding the overall interpretation of the data. The discussions primarily contributed to refinement of coding labels, level of detail, and the ongoing development of the coding scheme. Both researchers are experienced physiotherapists with clinical experience in rehabilitation and PCC; MNB has expertise in qualitative methodology, while AT had theoretical training but no prior practical experience. AT had been involved in recruiting and assessing participants in the preceding RCT, whereas MNB had no prior contact with any participants. AT’s prior involvement in the RCT provided contextual knowledge of the intervention but was also recognized as a potential source of pre-understandings during analysis. To enhance reflexivity, AT regularly discussed emerging codes and interpretations with MNB, who had no prior contact with participants, to challenge assumptions and support a balanced interpretation of the data.

All authors (AT, ASR, AS, UH, JB, ER, MNB), all of whom are women, contributed to the later interpretive stages, reviewing and discussing categories to ensure coherence and reach consensus. Authors included physiotherapists and a physician with experience in PCC and/or qualitative methods. One author (ASR) had previous participant contact during the RCT. Throughout data collection and analysis, the researchers reflected on and discussed how their professional backgrounds, experience of rehabilitation and PCC, and involvement in the RCT could influence data collection and interpretation, thereby supporting a balanced analytical process. These reflexive discussions were used to challenge assumptions and support a balanced analytical process.

Demographic data, medical history, and clinical characteristics were summarized using median and interquartile intervals (Q1–Q3) for continuous and ordinal variables, and n/N (%) for binary variables.

## 3. Results

### 3.1. Participants

Twelve of the 16 eligible individuals participated (Figure 1), and their demographic, medical history, and clinical characteristics are presented in Table 2. The four non-participants were generally similar to the interviewed participants, although they tended to be somewhat younger (range: 22–63 years), included a higher proportion of men (2/4), had higher baseline MIP values (range: 60–72% of predicted), and included two participants with postural orthostatic tachycardia syndrome (POTS).

Most participants were middle-aged women with higher education (>12 years) who had been physically active before contracting COVID-19, but reported reduced physical activity levels, severe fatigue, PESE, and respiratory symptoms at the time of entering the preceding RCT (Table 2). Interviews were conducted via video (*n* = 5), telephone (*n* = 5), or in person (*n* = 2), lasted 23–81 min (median 51), and were held a median of 46 days after RCT completion (Q1–Q3: 28–109).

### 3.2. Overview of Categories

Participants’ experiences were captured in five content categories that together described different aspects of the process of engaging in and completing the intervention. The categories reflected experiences related to entering the intervention, navigating and adapting over time, facing challenges during participation, receiving professional support, and perceived outcomes (Figure 2).

### 3.3. Finding Meaning and Feeling Ready

This category describes participants’ experiences of feeling ready and perceiving participation as meaningful, including expectations and goals, timing in relation to recovery, and the opportunity to take an active role in rehabilitation.

#### 3.3.1. Readiness to Engage

Expectations and goals ranged widely, from having none to anticipating full recovery or normalized breathing function and were often articulated in relation to physical aspects. Participants described entering the intervention with varying degrees of readiness, shaped by both physical recovery and previous rehabilitation experiences. Several recognized that participation might not have been possible at an earlier stage of recovery. As one participant put it:

At first, I thought, ‘If only I had been able to start this earlier, maybe I would have…’. But as we talked it through, I realized the timing was right. […] I might have wanted to jump into this two days after coming home, but I wouldn’t have managed. So, it really was the right moment.

(Participant 2)

#### 3.3.2. Gratitude for an Opportunity to Act

Several described feeling grateful for the opportunity to receive support and expressed an openness to try something that might improve their health. Receiving a tangible tool to act upon was perceived as valuable after an extended period of uncertainty. One participant expressed this clearly:

I felt that, now I’ve been given a tool to do something about this myself. So, I’m damn well going to go all in. If there is anything that can help, then I’m going to do it. I just felt that I couldn’t give up—I’m 45, I have a six-year-old and an eight-year-old; I can’t just give up and stop living. Because that’s what it becomes—if you can’t breathe, if you can’t get from point A to point B, it’s not only you who is affected; it affects the whole family. The breathing training gave me a tool to influence my situation myself. Mentally, that made it very important and very valuable.

(Participant 1)

### 3.4. Continuously Navigating and Adapting

This category captures participants’ ongoing process of learning, adjustments, and adaptations as they tailored the intervention to their symptoms and everyday circumstances.

#### 3.4.1. A Gradual Learning and Cautious Progression Process

Several participants described struggles with breathing technique during IMT. Mastering the technique involved a gradual learning process, with repeated practice described as helping to build confidence and improve performance. One participant illustrated this process by comparing it to relearning movement after long-standing compensations:

When I started the breathing exercise, it was difficult […] if you have osteoarthritis in one leg, you walk with a limp, and even after surgery, you keep limping because the body has forgotten how to use the leg. It’s the same with breathing, re-learning to breathe down into the stomach, then upward, and then out.

(Participant 5)

As confidence and familiarity increased, some participants described gradually increasing exercise load, IMT-resistance, or training volume, often starting cautiously and progressing as it felt more manageable. Monitoring exertion or resistance levels was described as helpful and as providing a sense of control.

#### 3.4.2. Individual Adaptions and Flexibility

Individual adaptations of the interventions were perceived as central. Participants emphasized the need to modify both physical exercise and IMT—by lowering resistance, changing position, or splitting sessions—often in collaboration with the physiotherapist. One participant described adapting IMT to their individual needs:

At first, there were ten repetitions three times, but it became too hard. In the end, I did five in the morning and five in the evening. […] I changed it to the lowest resistance. I never increased it again.

(Participant 3)

Participants also described flexibility in exercise settings as an important enabler, allowing them to perform both IMT and physical exercise at home, at work, or while travelling despite fluctuating symptoms. As one participant explained:

About the physical exercise, I do it at my own pace—whether I’m at the gym or somewhere else. If I’m very tired and don’t have the energy to go to the gym or can’t manage it, then I simply don’t go. But at home, for example, it’s easier… you’re more comfortable, and you have the equipment right there.

(Participant 7)

### 3.5. Facing Demanding and Symptom-Related Challenges

This category captures participants’ experiences of physical, emotional, symptom-related, and practical challenges encountered during and beyond the intervention period.

#### 3.5.1. Physical and Emotional Strain During Exertion

Initially, the interventions were often perceived as highly challenging. Participants described emotional and mental responses ranging from fear and uncertainty to determination. First impressions of IMT often included resistance levels that felt excessively heavy and heightened awareness of respiratory limitations.

It became very clear that I was extremely weak in my breathing muscles—I got it in black and white, and I hadn’t really understood that before […]. And I just thought, ‘God, am I the worst of all? I can’t even breathe in—what is this?

(Participant 1)

Participants described a range of physical sensations while performing the interventions. For IMT, breathing was perceived as physically demanding, often accompanied by dizziness, nausea, and a sense of relief when finishing the last breaths. Physical exercise was associated with feelings of heaviness, muscle soreness, fatigue, and an initial heightened awareness of physiological responses, such as an elevated heart rate, which some found unsettling. Some participants described the exercises as manageable, whereas others perceived them as strenuous.

#### 3.5.2. Symptom-Related Uncertainty

Several participants described how variations in symptom severity made planning and performance challenging, sometimes limiting activity or triggering caution. Fear of triggering PESE created additional uncertainty for some, and experiencing PESE interfered with activity and daily functioning as one participant clearly explained:

When I do things involving resistance, it’s not during the exercise itself, but afterwards… this tight ‘corset’ sensation kicks in, and it becomes difficult. Then it gets hard to breathe during the day. And when breathing is difficult, I get tired, which means I can’t do what I had planned.

(Participant 3)

#### 3.5.3. Personal and Practical Barriers to Engagement

Participants’ engagement with the interventions varied, and commitment was often linked to personal traits such as discipline and persistence. Some found the physical exercise program repetitive and wished for more variation or options that aligned with their preferences, while others expressed enjoyment and satisfaction.

Practical demands and life circumstances sometimes created stress and time constraints that challenged continuity, and some participants felt unprepared for the overall time commitment. While participants on full- or part-time sick leave found it easier to cope with this, those working full-time found it more challenging:

It probably depends on what kind of job you have, but I found it very difficult to have the time and energy to manage full-time work.

(Participant 8)

Some participants described continued engagement with IMT and physical exercise beyond the intervention period, motivated by perceived benefits and the prospect of future improvement. While some managed to establish regular routines with IMT or physical exercise, others practiced the interventions only occasionally or continued with other physical activities. Some participants described the absence of continued structured support as making it more difficult to maintain training independently, while others struggled due to symptoms and work demands.

### 3.6. Professional Support for Safety and Engagement

This category captures aspects of how professional support, including a trusting relationship, clinical expertise, and regular follow-up, was described as contributing to participants’ feelings of safety, confidence, and engagement in the intervention.

#### 3.6.1. A Trusting Relationship

Being met with empathy and respect, feeling listened to, and receiving responsive and individualized support were described as key aspects of professional support, which participants perceived as contributing to trust. Participants also emphasized that being monitored by knowledgeable professionals and having resources available when performing the interventions was perceived to contribute to a sense of safety. As one participant explained:

For me, it felt safe to train there. It gave me the confidence to push myself because I knew that if I fainted or if something felt wrong, there were resources available to help.

(Participant 3)

#### 3.6.2. Regular and Flexible Follow-Up as Key

Participants described the regular follow-up sessions as crucial for maintaining adherence, and descriptions of unplanned gaps in contact often led to reduced discipline. Scheduled follow-ups offered opportunities for practical guidance, detailed instructions, and clarification of uncertainties, while also providing accountability even on days of severe fatigue or low motivation. As one participant explained:

I think the regular contact was important, so that even on the days when you felt, ‘no, I just can’t manage this,’ you still pushed yourself to do it because you knew you would be called the following week. I do think that played a role in getting the training done.

(Participant 10)

A flexible format and scheduling, with the opportunity to choose between follow-ups via phone, video, or in-person visits, as well as to select preferred times to some degree, was valued for reducing practical barriers.

### 3.7. Divergent Perceptions of Intervention Effects

This category reflects participants’ varying and sometimes contrasting perceptions of intervention outcomes, ranging from partial improvements, including renewed trust in their bodies, to uncertainty, no perceived change, or setbacks.

#### 3.7.1. Partial Improvement and Renewed Bodily Trust

Perceived physical improvements included feeling stronger, breathing more easily, and experiencing less chest pressure and fatigue. For some participants, improvements in their individual test results from the preceding RCT reinforced their sense of progress. A few described positive effects on activities of daily living, such as unloading the dishwasher without needing to rest, sleeping more comfortably, walking longer distances, cycling to work, and managing stairs with greater ease. Some participants described that being able to do more than expected without experiencing setbacks helped them regain trust in their bodies and encouraged them to resume previously avoided activities. As one participant explained:

And then I felt safe again and dared to trust my body, and then I dared to take my walks […] I’ve even run 100 m, but I don’t feel safe enough to do more than that because it becomes too difficult.

(Participant 2)

While some participants described improvement, they emphasized that they had not returned to their pre-COVID levels of functioning. The need for breaks, persistent symptoms, and continued day-to-day fluctuations meant that recovery was perceived as partial rather than complete:

They aren’t gone, but I can cycle, I can talk while I’m cycling, I can walk and talk. On days that aren’t so good, I still feel more limited by the pain in my legs than by breathlessness. It doesn’t restrict me as much anymore.

(Participant 9)

#### 3.7.2. No Change, Uncertainty or Setbacks

Several participants expressed uncertainty about whether the intervention had resulted in meaningful changes. They found it difficult to distinguish intervention-related effects from the natural fluctuations of PCC symptoms and reported little noticeable change in breathing difficulties, fatigue, or everyday functioning. Others questioned whether perceived improvements reflected actual recovery, explaining that any changes were often subtle and difficult to identify in daily life. Some also described setbacks during the intervention period, including worsening of existing symptoms, the development of new health problems, or follow-up test results indicating deterioration despite sustained engagement in the intervention:

Well, I don’t know. It’s difficult to say whether this has helped me. I made an effort to do the exercises, but whether it has helped me in any way, I can’t really say. I don’t think it was particularly helpful.

(Participant 11)

## 4. Discussion

Adults with PCC and inspiratory muscle weakness who were able to participate in an individualized intervention combining IMT with physical exercise entered the intervention with varying expectations, described a sense of readiness to participate, and expressed gratitude for the opportunity to take part. They perceived the intervention as a meaningful and timely opportunity for self-directed action in managing their health. Managing challenging exertion, symptom-related uncertainty, and everyday demands involved learning, individual tailoring, and flexibility in where training was performed. Professional support was described as contributing to a perceived sense of safety and supporting continued engagement through trusting relationships and regular follow-up. Perceived outcomes varied considerably. While some participants described partial physical improvement and renewed trust in the body, others reported uncertainty regarding benefits, no noticeable change, or setbacks.

Our findings are consistent with previous studies in PCC showing that individuals experience IMT as an effective intervention that provides a tool for self-management [13,14]. The central importance of professional support, both for adherence and for assistance with technique, also echoes prior work [12,13,14]. We similarly found the need to individually adjust the number of repetitions and sets in IMT and to apply flexibility in scheduling to accommodate fluctuating symptoms [12,13]. In line with earlier studies on experiences of physical activity and exercise in PCC, we also found that individuals described experiencing PESE, which required flexible support and adaptation of activity or exercise dose [15,16,17]. Unlike previous studies that explored IMT or physical exercise in isolation, our findings illustrate how a tailored intervention combining IMT and physical exercise is experienced within the context of everyday life. Participants not only navigated symptom variability and concerns about symptom exacerbation but also balanced rehabilitation with work, family responsibilities, and other daily demands. These findings highlight the importance of flexible, individually tailored interventions that can be integrated into everyday life and adapted to changing circumstances and individual needs. This study therefore provides a more nuanced understanding of how participants experience and adapt a combined exercise-based intervention in practice.

The experiences described demonstrate that rehabilitation for PCC must be understood within the context of a highly fluctuating and complex symptom trajectory. Day-to-day symptom variation, PESE, uncertainty about bodily responses, and competing everyday demands shaped how participants approached both inspiratory muscle training and physical exercise, where emotional, cognitive, and physical responses were tightly interwoven. The findings on symptom-related uncertainty and professional support may be viewed through the lens of Mishel’s Uncertainty in Illness Theory, which suggests that unpredictability and difficulties interpreting symptoms may hinder engagement in rehabilitation unless adequate support is provided to help individuals make sense of their experiences [28]. Participants described uncertainty related not only to symptom fluctuations and PESE, but also to how much exertion was safe and manageable. Within this context, professional support and the structured intervention appeared to help participants gradually interpret bodily responses and engage more confidently in rehabilitation.

The intervention was also perceived as offering a tangible opportunity to influence one’s own recovery and take an active role in managing health challenges. Self-efficacy theory may offer one perspective for interpreting this finding, proposing that experiences of successfully performing meaningful health-related actions can strengthen confidence in one’s ability to manage health challenges [29]. Participants’ descriptions of learning to adapt training, monitor symptoms, and gradually increase activity suggest that the intervention may have supported such self-efficacy processes in both rehabilitation and everyday life.

The importance of reducing uncertainty and supporting confidence in managing symptoms further underscores the need for a person-centered rehabilitation approach. Individual tailoring emerged as essential, described by the ongoing need for adjustments to intensity, pacing, position, and setting, while also taking psychological and contextual needs into account [30]. These insights reinforce that flexible, individualized delivery is not an optional feature but a central component of rehabilitation for individuals living with PCC.

Participants valued both trusting relationships with knowledgeable professionals and regular, flexible follow-up that supported engagement, aligning with known determinants of adherence such as therapeutic alliance and the importance of addressing perceived barriers and providing consistent feedback [31,32]. Although the improvements described were perceived as partial rather than complete, many participants described symptom relief and increased physical function, along with renewed confidence in their physical abilities. These findings suggest that the value of the intervention may extend beyond physiological improvements. Participants’ descriptions suggest that the intervention was perceived as supporting engagement in activity, symptom management, and experiences of mastery, and may have contributed to renewed trust in their bodies and confidence in managing everyday life, consistent with key principles of self-efficacy theory [29].

Rehabilitation services for PCC and inspiratory muscle weakness should prioritize flexible models that incorporate individual adjustments, support multiple delivery settings, address symptom-related uncertainty and practical barriers to engagement, and offer regular patient-preferred follow-up. Programs should explicitly address PESE through pacing strategies and outcome assessment should combine physiological and patient-reported measures. Given the heterogeneity of PCC symptoms and responses to rehabilitation, careful individual assessment and selection of appropriate rehabilitation approaches are essential.

Future research should investigate which individuals with PCC are most likely to benefit from exercise-based rehabilitation, how interventions can be tailored to different symptom profiles and levels of symptom burden, and which components of professional support contribute most to adherence, self-efficacy, and perceived benefit. Mixed-methods designs may be particularly valuable for integrating clinical outcomes with participants’ perspectives. In addition, incorporating the perspectives of individuals who are unable to complete exercise-based interventions is essential for identifying barriers, unmet needs, and factors that may inform the selection and tailoring of rehabilitation approaches for different individuals with PCC.

### Methodological Considerations

The findings of this study should be interpreted in light of its limitations. Regarding the sample, only participants who had enrolled in and completed the preceding RCT were invited to take part in the interviews, and not all eligible participants chose to participate. Consequently, the experiences of individuals who were unable to complete the RCT, for example due to severe PESE, are not represented. In addition, although the four eligible participants who did not participate in the interviews were broadly similar to those interviewed with respect to demographic, medical history, and clinical characteristics, they tended to be somewhat younger, were more often male, more frequently had POTS, and had higher baseline inspiratory muscle strength. This may have introduced selection bias, as participants who completed both the intervention and the interviews may have had more favorable experiences of, or greater tolerance of, the intervention than those who withdrew or declined interview participation. Consequently, the findings may primarily reflect the experiences of individuals who are able to engage in exercise-based rehabilitation interventions and may portray engagement, safety, and perceived benefits more positively than would have been the case had the perspectives of non-completers been represented. Moreover, participants were recruited from a single tertiary-level service, and most were middle-aged women with a high level of education. Although this participant profile is broadly consistent with populations commonly encountered in PCC clinical practice and research in similar settings, the findings may be less transferable to men, younger or older individuals, people with other educational backgrounds, individuals with POTS or severe PESE, and individuals receiving care in other settings.

Nevertheless, sample adequacy was judged based on the relevance of participants’ experiences to the study aim and the variation in demographic and clinical characteristics represented in the sample, consistent with recommendations regarding trustworthiness and sampling in qualitative content analysis [33]. All participants who met the eligibility criteria were invited to participate in the interview study. The participants represented variation in demographic and clinical characteristics, enabling the exploration of a range of perspectives relevant to the study aim. The interviews provided rich and varied accounts, including both recurring patterns and divergent experiences, supporting the development of nuanced categories describing different aspects of participants’ experiences.

The use of two interviewers across different time periods, as well as variation in interview mode (onsite, telephone, or video) and in the timing of the interview after completing the intervention, may have introduced inconsistency in data collection. Participants interviewed longer after intervention completion may have had greater difficulty recalling specific experiences, while variations in interview modality may have influenced opportunities for interviewers to identify cues for further probing and exploration, potentially affecting the depth and nuance of the data obtained. However, no apparent differences in data richness, depth, or nuance were observed in relation to interview modality or the time elapsed between intervention completion and the interview, although subtle influences cannot be ruled out. The semi-structured interview guide supported consistency in data collection, and flexible interview formats were considered a strength, as they enabled participants to choose the mode they preferred and facilitated participation by reducing practical barriers to involvement for participants experiencing severe fatigue and PESE. Although the interviewers had no prior relationship with participants and were not involved in the RCT, social desirability bias cannot be excluded, as participants may have been inclined to emphasize positive experiences.

Individual interviews and inductive qualitative content analysis were considered appropriate with the ambition to obtain rich, nuanced descriptions of participants’ experiences within a specific context [19]. Although participants sometimes reflected on specific aspects of IMT or physical exercise, the subcategories and categories were developed to reflect experiences of the combined intervention and its delivery, consistent with the study aim. The subcategories and categories were generally derived from participants’ accounts across multiple interview topics rather than from responses to individual interview questions. The analysis intentionally remained close to the manifest content, focusing on meaning as inferred from the immediate context, which was considered appropriate given the aim of describing participants’ experiences of a specific rehabilitation intervention [25]. Several strategies were used to strengthen the quality of the study, including the enrolment of all eligible participants, use of a semi-structured interview guide, partial independent coding followed by interpretive discussions among multiple researchers, documentation of analytic decisions through memos, and the use of representative quotations. Although transcripts were not returned to participants for comment or correction, and participants did not provide feedback on the findings, credibility was supported through clarifying questions and reflective summaries during interviews to verify understanding of participants’ accounts. Acknowledgment of authors’ pre-understandings and clear descriptions of context, participants, procedures, and analytical steps further enhanced transparency [33]. The researchers’ clinical experience in rehabilitation and PCC may have facilitated understanding of participants’ accounts and the clinical context, while also creating a risk that interpretations could be influenced by existing professional assumptions.

## 5. Conclusions

Adults with PCC and inspiratory muscle weakness who were able to participate in an intervention combining IMT with physical exercise described both benefits and challenges, and often perceived it as a meaningful opportunity to take an active role in their recovery. Individual tailoring, flexible delivery, and professional support were described as supporting engagement despite challenges with symptom fluctuation and PESE, as well as exertional and everyday demands. Perceived outcomes ranged from setbacks and no noticeable change to partial improvement, including renewed trust in the body. These findings highlight the importance of individualised rehabilitation approaches that incorporate ongoing support and adaptation to fluctuating symptoms. Such approaches may be perceived as valuable by individuals with PCC who are able to engage in exercise-based interventions.

## Figures and Tables

**Figure 1:**
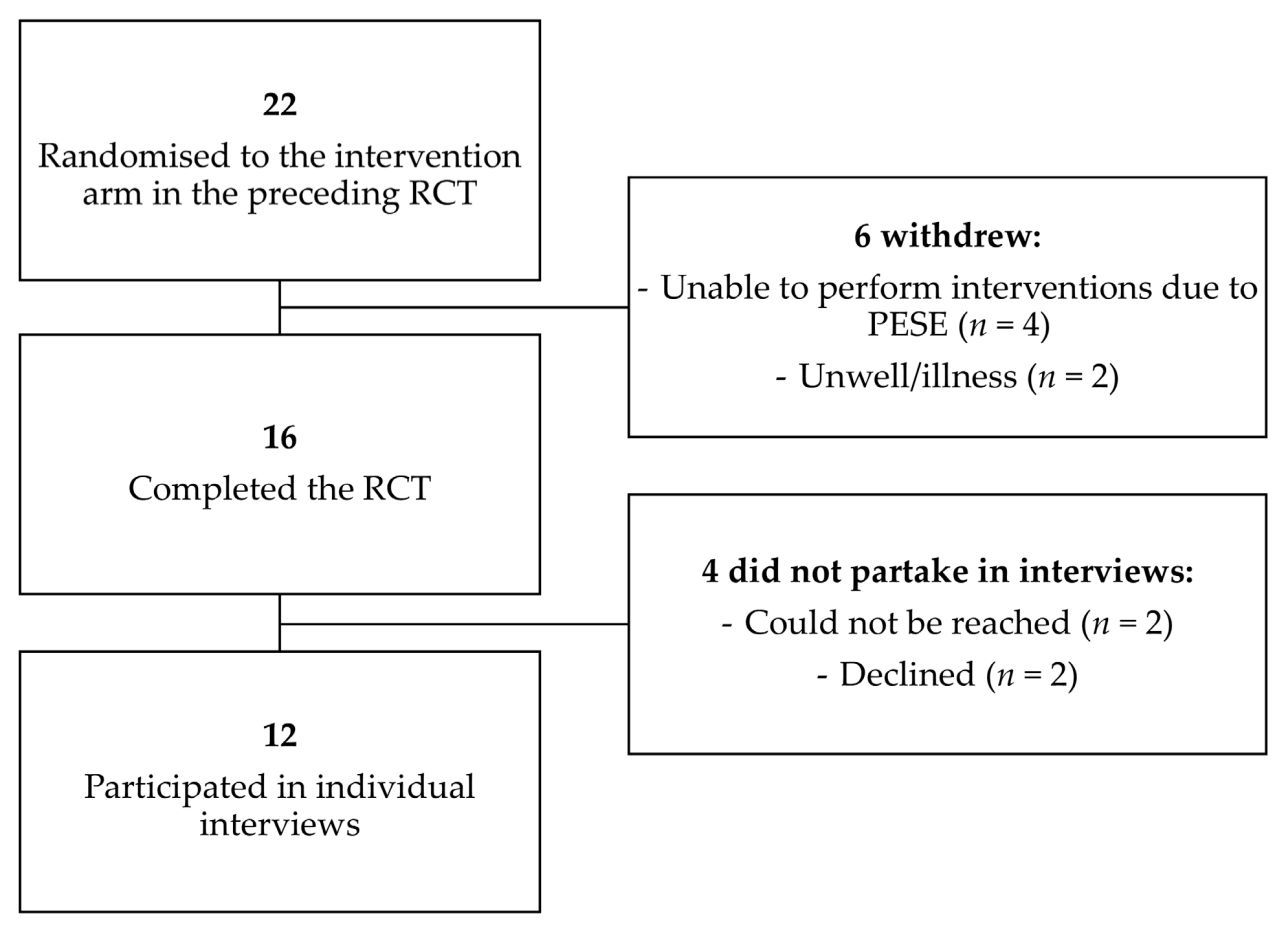
Flowchart of participants, showing the number randomized to the intervention arm in the preceding RCT, completing the trial, and participating in individual interviews.

**Figure 2:**
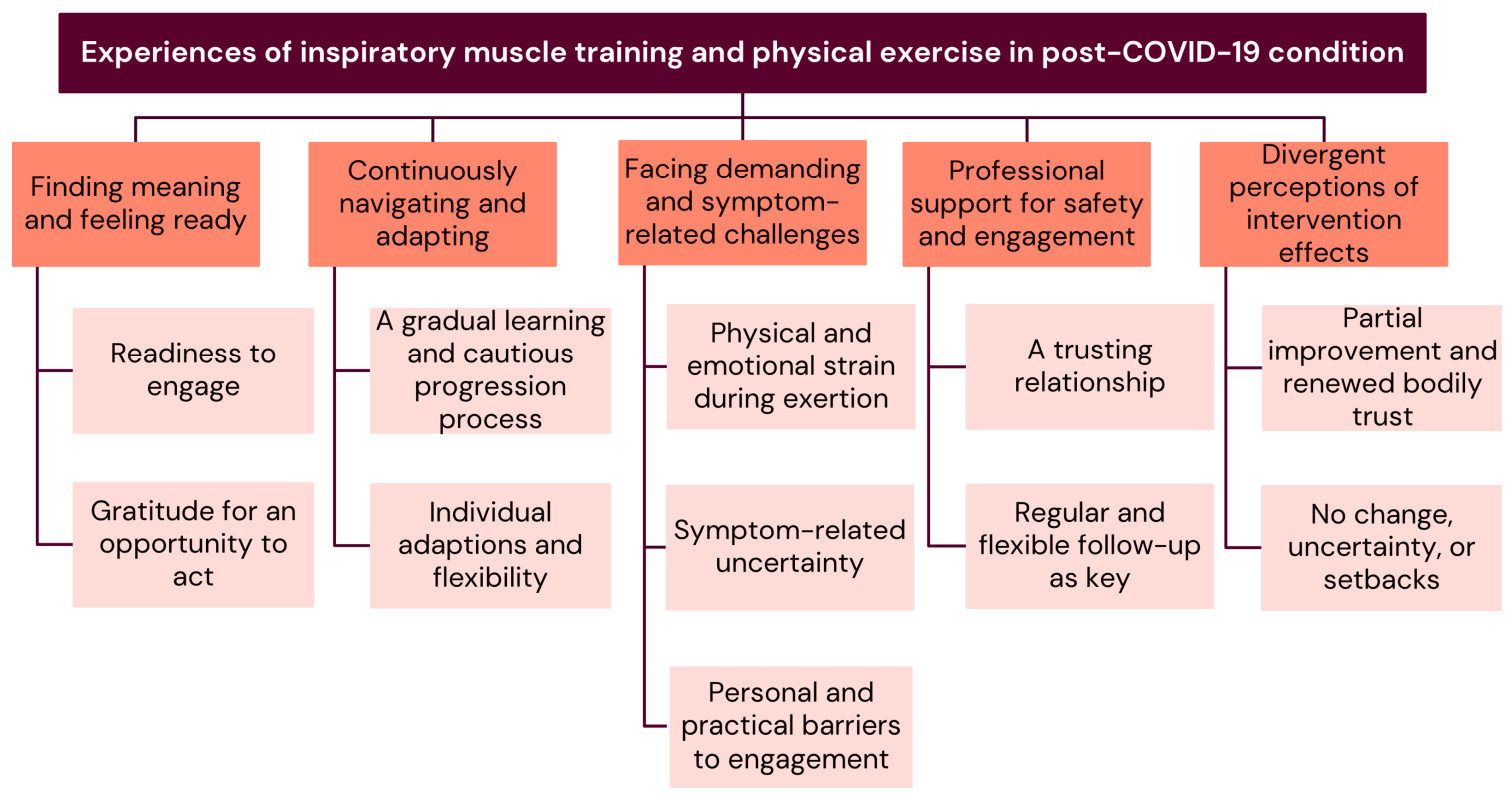
Overview of the five content categories and subcategories reflecting the experiences of adults with post-COVID-19 condition and inspiratory muscle weakness participating in an individualized intervention combining inspiratory muscle training and physical exercise.

**Table 1:** Examples of the coding process from meaning units to codes, subcategories, and content categories.

Meaning Unit	Code	Subcategory	Content Category
“It was really scary because it made it very clear just how extremely weak my breathing muscles were.”	First impression of IMT	Physical and emotional strain during exertion	Facing demanding and symptom-related challenges
“I probably did it a bit too quickly just to get it over with. By the end, I was doing the exercises correctly.”	Mastering IMT technique	A learning and cautious progression process	Continuously navigating and adapting
“For me, it felt reassuring to be there and exercise. It made me feel confident enough to push myself because I knew that if I fainted or if something felt wrong, support was available.”	Feeling safe	A trusting relationship	Professional support for safety and engagement

**Table 2:** Demographic, medical history, and clinical characteristics assessed at baseline of the preceding RCT among interview participants with post-COVID-19 condition and inspiratory muscle weakness (*n* = 12). Data are presented as median (Q1–Q3) or *n* (%).

Variable	Value
*Demographics*	
Age, years	54 (39–64)
Female sex	11 (92%)
Higher education (>12 years)	10 (83%)
Current employment status:	
Sick leave > 50%	4 (33%)
Work/Study > 50%	6 (50%)
Retired	2 (17%)
*Medical history*	
Never smoker	11 (92%)
Pre-COVID-19 self-reported physical activity:	
Frändin–Grimby activity score (0–6)	5 (4–6)
Number of co-morbidities	1 (1–2)
Asthma	1 (8%)
COPD or Interstitial Lung Disease	0 (0%)
Postural Orthostatic Tachycardia Syndrome	0 (0%)
Hospitalised due to COVID-19	4 (33%)
Intensive care unit	2 (17%)
*Clinical characteristics (at baseline of preceding RCT)*	
Severe fatigue (FSS ≥ 4)	11 (92%)
Severe dyspnea (mMRC ≥ 2)	12 (100%)
Self-reported symptoms (present/absent):	
PESE	10 (83%)
Difficulties taking deep breaths	10 (83%)
Chest tightness	10 (83%)
Inspiratory muscle strength (MIP), % of pred	49 (47–57)
Self-rated health (EQ VAS), (0–100)	55 (40–70)
Self-reported physical activity:	
Frändin–Grimby activity score (0–6)	3 (3–4)

Abbreviations: COPD: Chronic Obstructive Pulmonary Disease; FSS: Fatigue Severity Scale; MIP: Maximal Inspiratory Pressure; mMRC: modified Medical Research Council dyspnea scale; PESE: Post-Exertional Symptom Exacerbation; RCT: Randomized Controlled Trial.

## Data Availability

Complete interview transcripts cannot be shared as they may compromise participant confidentiality. Other data are available upon reasonable request via the Research Data Office at Karolinska Institutet (rdo@ki.se) and will be provided in accordance with applicable legislation and institutional requirements. Access may require a data processing agreement or similar arrangement with the recipient institution.

## Supplementary Materials

The following supporting information can be downloaded at https://www.mdpi.com/article/10.3390/jcm15197414/s1, S1: Consolidated criteria for reporting qualitative studies (COREQ): 32-item checklist; S2: Interview guide.

## Abbreviations

The following abbreviations are used in this manuscript:

COPD	Chronic Obstructive Pulmonary Disease
EQ VAS	EuroQol Visual Analog Scale
FSS	Fatigue Severity Scale
IMT	Inspiratory Muscle Training
MIP	Maximal Inspiratory Pressure
mMRC	Modified Medical Research Council Dyspnea Scale
PCC	Post-COVID-19 Condition
PESE	Post-Exertional Symptom Exacerbation
POTS	Postural Orthostatic Tachycardia Syndrome
Q1	First Quartile
Q3	Third Quartile
RCT	Randomized Controlled Trial
ReCOV	Recovery and Rehabilitation During and After COVID-19
