# Supplementary material for: Experiences of Inspiratory Muscle Training and Physical Exercise in Adults with Post-COVID-19 Condition and Inspiratory Muscle Weakness: A Qualitative Study
Source: J Clin Med. 2026 Sep 24;15(19):7414. doi: 10.3390/jcm15197414 (PMC13666434; doi:10.3390/jcm15197414)
Supplement: Supplementary file 1 [file jcm-15-07414-s001.zip › Supplementary materials_S2_Interview Guide.pdf]

Interview Guide – Question Areas

Information beforehand
Purpose of the study, confidentiality, voluntary participation, and that there are no right or wrong answers.

Respiratory symptoms
Experiences of respiratory symptoms after COVID-19, including any persistent or limiting symptoms.

Experiences of performing the intervention
Overall impressions of the intervention; early versus later experiences; changes over time; perceived challenges; and symptom responses during or after training.

Perceived safety and security
Sense of safety when performing the training and factors influencing this.

Perceived helpfulness or lack of effect
Situations where the intervention felt beneficial or unhelpful; thoughts about continuing or discontinuing.

Structure and content of the intervention
Reflections on duration, frequency, intensity, pacing and overall demands, and reasons behind these perceptions.

Follow-up with the physiotherapist
Experiences of the organisation and format of follow-up visits (on-site/remote), perceived support, and views on the set-up.

Expectations and personal goals
Participants' expectations and individual goals related to rehabilitation.

Perceived meaningful aspects
What participants found most valuable or important in the rehabilitation.

Perceived effects and impact
Improvements or setbacks affecting daily life, work, fatigue management, leisure activities, family life, and overall functioning.

Support needs
Support received from professionals, family, friends, or peers, and support participants felt they needed.

Suggestions or unmet needs
Views on how the rehabilitation could have been organised differently or what was missing
